# Pituitary Apoplexy and Subdural Hematoma after Caesarean Section

**DOI:** 10.1155/2022/3097949

**Published:** 2022-06-23

**Authors:** Van Trung Hoang, The Huan Hoang, Thanh Tam Thi Nguyen, Vichit Chansomphou, Duc Thanh Hoang

**Affiliations:** ^1^Department of Radiology, Thien Hanh Hospital, Buon Ma Thuot, Vietnam; ^2^Department of Radiology, FV Hospital, Ho Chi Minh City, Vietnam; ^3^Department of Radiology, Savannakhet Medical-Diagnostic Center, Kaysone Phomvihane, Laos; ^4^Department of Endocrinology, Diabetes and Metabolism, Walter Reed National Military Medical Center, Bethesda, USA; ^5^Department of Medicine, Uniformed Services University of the Health Sciences, Bethesda, USA

## Abstract

Pituitary apoplexy can occur postpartum, and subdural hematoma following epidural anesthesia is a rare complication. Cooccurrence of these two complications is extremely rare and has not been previously reported in the literature. In this article, we present a case of pituitary apoplexy along with intracranial subdural hematoma happening two days after spinal anesthesia for cesarean section. The patient presented with peripheral facial nerve paralysis accompanied by headache, eye pain, and blurred vision and was diagnosed by imaging modalities. The patient made a good recovery with conservative treatment without serious health events.

## 1. Introduction

Epidural anesthesia and spinal anesthesia to assist in obstetric procedures have become the current standard techniques with proven safety and efficacy. However, anesthesia and caesarean section may cause some certain complications such as intracranial bleeding that can be potentially lethal. Few cases of intracranial subdural hematoma following the administration of epidural anesthesia have been reported in the literature [1–5]. Very few cases of pituitary apoplexy have been reported in postpartum patients [6–10]. To our knowledge, the cooccurrence of pituitary apoplexy and subdural hematoma after spinal anesthesia for cesarean section has not been reported before. We report a rare case where multiple intracranial complications occurred simultaneously after a cesarean section using spinal anesthesia with the presence of symptoms of peripheral facial paralysis. The patient was treated conservatively and recovered well thereafter.

## 2. Case Presentation

**Figure 1:**
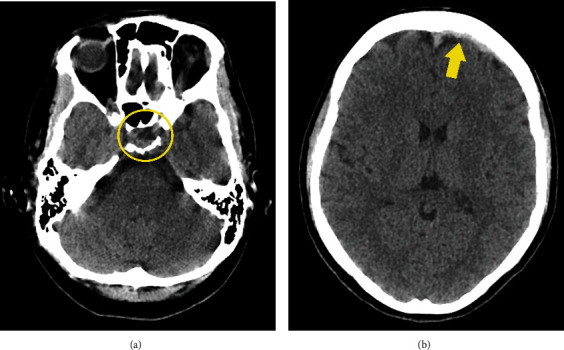
(a) Axial CT of pituitary apoplexy appearance indicates heterogeneous hyperattenuating intrasellar lesion due to the presence of hemorrhagic components with a fluid debris level (circle). (b) Axial CT image shows a thin layer of acute subdural hematoma in the left frontal region (arrow).

**Figure 2:**
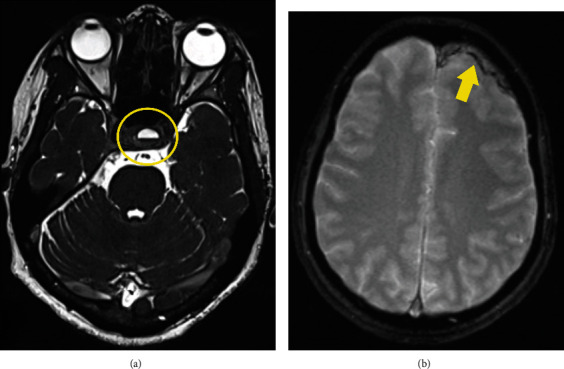
(a) CISS 3D MRI image shows the level of fluid debris in the pituitary fossa (circle). (b) T2∗-weighted gradient-echo MRI image shows the signal intensity of a subdural hematoma (arrow).

A 34-year-old primigravida at 38 weeks gestation starting labor was hospitalized. She had a benign tumor in the left ovarian which had been operated on 2 years ago. Cardiotocography showed category II, and clinical examination manifested cephalopelvic disproportion, so cesarean section was indicated. The baby was delivered through cesarean section by the Pfannenstiel line under spinal anesthesia. After the surgery, the patient recovered within normal limits. After two days of surgery, she showed signs of right facial paralysis. Her chief complaints were headaches, mild eye pain, and blurred vision. She was normotensive with a blood pressure of 110/60 mmHg, pulse rate 100 bpm, respiratory rate 21 per minute, and temperature 37°C. Physical examination revealed mild right-sided facial and mouth distortion (drooping). Otherwise, no other focal neurological signs were found. Laboratory results performed before and after cesarean section in this case including a complete blood count, coagulation, blood glucose, liver and kidney function, TSH, and free T4 and T3 were within normal ranges. She underwent a head computed tomography (CT) scan showing mildly hyperattenuating pituitary gland and thin layer subdural hematoma in the left frontal region (Figure 1). A subsequent head magnetic resonance imaging (MRI) revealed pituitary apoplexy and left frontal subdural hematoma (Figure 2). The patient was treated conservatively with the usual supportive measures such as vital sign monitoring, airway support, intravenous fluids, and nutritional support. She did not need hormone imbalance or surgery. Her symptoms were relieved, and she was discharged after 7 days. She continued doing well at one-year follow-up. Laboratory tests and imaging exams to check pituitary function did not reveal any abnormalities.

## 3. Discussion

Epidural anesthesia and spinal anesthesia are frequently used in obstetric procedures; however, they can cause complications such as back pain, headache, hemorrhage, infection, nerve injury, and intracranial hematoma. Intracranial subdural hematoma is a rare complication but can lead to life threatening [11]. The clinical appearance of a subdural hematoma includes persistent headache, vomiting, drowsiness, disorientation, blurring of vision, and other neurological symptoms [12]. Pituitary apoplexy is defined as acute nonhemorrhagic or hemorrhagic infarction of the pituitary gland. The risk of pituitary hemorrhage and infarction is increased during pregnancy and the postpartum period. Pituitary apoplexy occurring after spinal anesthesia for cesarean sections is an extremely rare acute clinical condition that presents with symptoms including sudden headache, nausea, vomiting, visual disturbances, meningeal signs, and altered consciousness [13, 14]. However, the symptoms are the combined results of subdural hematoma and pituitary apoplexy as such they may overlap each other [1, 2, 6, 10].

These situations can be explained by several pathogenesis. First, for subdural hematoma, the primary mechanism leading to it after spinal anesthesia is intracranial hypotension that causes a caudal shift of the brain. This leads to traction of broken dural vessels (such as a small cerebral cortical vein, the bridging dural vein, or dural venous sinus wall) that result in blood extravasation and the formation of subdural hematoma [2, 15].

Second, for pituitary apoplexy, a mechanism may be explained by the increased volume of the pituitary gland in pregnant women due to lactotroph cells undergoing massive hyperplasia. Estrogen receptors are expressed in the lactotroph cells, and estrogen levels become very high during pregnancy. Even though lactotroph cells become larger, both in the pituitary gland and the pituitary adenoma, the blood supply remains limited leading to pituitary stroke [10, 13, 16].

Another possible mechanism for pituitary apoplexy in this circumstance can be the subacute, excessive growth of the preexisting adenoma, which outgrows its blood supply with eventual ischemic necrosis followed by hemorrhage. Some causes of pregnancy-related pituitary apoplexy include intracranial hypertension, diabetes, dynamic pituitary testing, bromocriptine, and anticoagulants. Identified risk factors are tumor factors including histological type, nonfunctioning tumor or prolactinoma, and size (macroadenoma), along with patient factors such as pregnancy, systemic hypertension, dopamine agonist administration, dynamic pituitary function tests, and anticoagulant agents. Indeed, pathologic and dynamic imaging studies have shown that macroadenomas, as well as microadenomas, are less vascularized than the pituitary gland so that relatively fast and sizable growth can exceed this low blood supply. However, this theory does not explain the onset of pituitary apoplexy in patients with small adenomas or with a healthy pituitary [6, 13, 14, 16].

Another hypothesis is that tumor compression of the infundibulum and superior pituitary arteries may cause infarction of the normal pituitary gland; however, ischemia of the tumor mass itself is less probable in this case because the vessels supplying the adenoma are attributable to the inferior pituitary circulation. Therefore, pituitary tumors probably suffer from an intrinsic vasculopathy that can lead to spontaneous infarction and hemorrhage [13, 16].

Sheehan syndrome is a typical obstetric-related inclined factor and a rare cause of pituitary apoplexy as well as panhypopituitarism. It occurs only in women who suffered a severe postpartum hemorrhage with the critical hypovolemic shock resulting in ischemic pituitary necrosis. Sheehan syndrome should be suspected in the case of persistent hypotension and tachycardia after severe obstetric hemorrhage treatment. Other premature signs of Sheehan syndrome are related to hypopituitarism, such as hypoglycemia and breastfeeding difficulty or inability [17].

Typical imaging features of intracranial subdural hematoma are generally changed in the cases happening after spinal anesthesia for cesarean section from a small hematoma to a large hematoma leading to a mass effect. CT and MRI are easy to diagnose intracranial subdural hematoma [18]. In our case, the subdural hematoma is presented as a high-density thin layer in the left frontal region. Radiological manifestations of pituitary apoplexy vary depending on the duration of apoplexy. A large pituitary gland can be observed in most cases. CT images can show patchy or confluent areas of hyperdensity in a pituitary lesion if a hemorrhagic component is present. The density of the lesion is often mixed and varies over time due to hemolysis. MRI may identify the presence of an adenoma and hemorrhagic degeneration of pituitary apoplexy lesions. Early findings appear as sellar enlargement and abnormal signal intensity with poor enhancement or rim enhancement. Late findings appear with an empty sella of normal size [13, 14, 19, 20].

Immediate medical treatment of subdural hematoma and pituitary apoplexy should begin with careful assessment of the patient's condition, management of the airway, and supportive measures to ensure hemodynamic stability, fluid, and electrolyte balance. After monitoring and stabilization of the patient, a secondary care plan should be implemented. Conservative nonsurgical management for subdural hematoma may be considered if the accumulation does not cause impingement on the brain or brain stem. Surgical evacuation should be performed promptly if there are signs of increased intracranial pressure threatening vital functions [21, 22]. Hypopituitarism can resolve on its own in some cases; in others, it will lead to irreversible hormone deficiencies. Hormone replacement therapy must be individualized to the needs of each specific patient. Patients may need lifelong hormone replacement therapy [23–25].

## 4. Conclusion

The coexistence of multiple intracranial complications is uncommon after spinal anesthesia for cesarean section. However, in the specific case, no connection exists between subdural hematoma, connected with anesthesia, and pituitary apoplexy, which specifically has no diagnosed cause. In short, it is a casual association. This case helps to add knowledge to the current literature and may be useful to clinicians managing patients if similar situations arise. It is essential to diagnose these conditions early and manage appropriately.

## Data Availability

There are no data to share. All the details have been mentioned in the manuscript.

## References

[1] Schweiger V., Zanconato G., Lonati G., Baggio S., Gottin L., Polati E. (2013). Intracranial subdural hematoma after spinal anesthesia for cesarean section. *Case Reports in Obstetrics and Gynecology*.

[2] Haase R., Kursawe I., Nagel F., Sitka U., Burdach S. (2003). Acute subdural hematoma after caesarean section: a case report. *Pediatric Critical Care Medicine*.

[3] Bi Y., Zhou J. (2021). Spinal subdural hematoma and subdural anesthesia following combined spinal-epidural anesthesia: a case report. *BMC Anesthesiology*.

[4] Gioia S., Mirtella D., Lancia M., Suadoni F., Cingolani M. (2019). Fatal acute intracranial subdural hematoma after spinal anesthesia for cesarean delivery: case report and review of the literature. *The American Journal of Forensic Medicine and Pathology*.

[5] Bekele D., Bayable M., Bedane A. (2021). Chronic intracranial subdural hematoma after spinal anesthesia for a cesarean section: a case report. *Journal of Medical Case Reports*.

[6] Couture N., Aris-Jilwan N., Serri O. (2012). Apoplexy of a microprolactinoma during pregnancy: case report and review of literature. *Endocrine Practice*.

[7] Geissler F., Hoesli I., Todesco B. M. (2021). Recurrent pituitary apoplexy in pregnancy. *BML Case Reports*.

[8] Chan J. L., Gregory K. D., Smithson S. S., Naqvi M., Mamelak A. N. (2020). Pituitary apoplexy associated with acute COVID-19 infection and pregnancy. *Pituitary*.

[9] Oguz S. H., Soylemezoglu F., Dagdelen S., Erbas T. (2020). A case of atypical macroprolactinoma presenting with pituitary apoplexy during pregnancy and review of the literature. *Gynecological Endocrinology*.

[10] Hayes A. R., O'Sullivan A. J., Davies M. A. (2014). A case of pituitary apoplexy in pregnancy. *Endocrinology, Diabetes & Metabolism Case Reports*.

[11] Moradi M., Shami S., Farhadifar F., Nesseri K. (2012). Cerebral subdural hematoma following spinal anesthesia: report of two cases. *Case reports in medicine*.

[12] Cantais E., Behnamou D., Petit D., Palmier B. (2000). Acute subdural hematoma following spinal anesthesia with a very small spinal needle. *Anesthesiology*.

[13] Boellis A., di Napoli A., Romano A., Bozzao A. (2014). Pituitary apoplexy: an update on clinical and imaging features. *Insights Into Imaging*.

[14] Goyal P., Utz M., Gupta N. (2018). Clinical and imaging features of pituitary apoplexy and role of imaging in differentiation of clinical mimics. *Quantitative Imaging in Medicine and Surgery*.

[15] Iwase Y., Suzuki M., Bito H. (2017). A case report of intracranial hemorrhage after spinal anesthesia. *JA Clin Rep.*.

[16] Rogg J. M., Tung G. A., Anderson G., Cortez S. (2002). Pituitary apoplexy: early detection with diffusion-weighted MR imaging. *AJNR. American Journal of Neuroradiology*.

[17] Karaca Z., Laway B. A., Dokmetas H. S., Atmaca H., Kelestimur F. (2016). Sheehan syndrome. *Nature Reviews Disease Primers*.

[18] Cuypers V., Van de Velde M., Devroe S. (2016). Intracranial subdural haematoma following neuraxial anaesthesia in the obstetric population: a literature review with analysis of 56 reported cases. *International Journal of Obstetric Anesthesia*.

[19] Miljic D., Pekic S., Popovic V. (2000). *Empty sella*.

[20] Muthukumar N. (2020). Pituitary apoplexy: a comprehensive review. *Neurology India*.

[21] Lim G., Zorn J. M., Dong Y. J., DeRenzo J. S., Waters J. H. (2016). Subdural hematoma associated with labor epidural analgesia: a case series. *Regional Anesthesia and Pain Medicine*.

[22] Amorim J. A., Remigio D. S., Damazio Filho O., de Barros M. A., Cervalho V. N., Valenca M. M. (2010). Hematoma subdural intracraneal postanestesia subaracnoidea: relato de dos casos y revision de 33 casos de la literatura. *Revista Brasileira de Anestesiologia*.

[23] Kim S. Y. (2015). Diagnosis and treatment of hypopituitarism. *Endocrinol Metab (Seoul).*.

[24] Fleseriu M., Hashim I. A., Karavitaki N. (2016). Hormonal replacement in hypopituitarism in adults: an endocrine society clinical practice guideline. *The Journal of Clinical Endocrinology and Metabolism*.

[25] Kato Y., Ogawa Y., Tominaga T. (2021). Treatment and therapeutic strategies for pituitary apoplexy in pregnancy: a case series. *Journal of Medical Case Reports*.

